# Prediction Model Based on DoE and FTIR Data to Control Fast Setting and Early Shrinkage of Alkaline-Activated Slag/Silica Fume Blended Cementitious Material

**DOI:** 10.3390/ma16114104

**Published:** 2023-05-31

**Authors:** Tim Schade, Bernhard Middendorf

**Affiliations:** 1 Department of Structural Materials and Construction Chemistry, University of Kassel, Moenchebergstr. 7, 34125 Kassel, Germany; 2 Deutscher Beton- und Bautechnik-Verein e.V., 10785 Berlin, Germany; schade@betonverein.de

**Keywords:** alkali-activated materials, FTIR, shrinkage, design of experiments

## Abstract

This study aims to develop a material-saving performance prediction model for fast-hardening alkali-activated slag/silica fume blended pastes. The hydration process in the early stage and the microstructural properties after 24 h were analyzed using design of experiments (DoE). The experimental results show that the curing time and the FTIR wavenumber of the Si-O-T (T = Al, Si) bond in the band range of 900–1000 cm^−1^ after 24 h can be predicted accurately. In detailed investigations, low wavenumbers from FTIR analysis were found to correlate with reduced shrinkage. The activator exerts a quadratic and not a silica modulus-related conditioned linear influence on the performance properties. Consequently, the prediction model based on FTIR measurements proved to be suitable in evaluation tests for predicting the material properties of those binders in the building chemistry sector.

## 1. Introduction

In the construction chemistry sector, binders with a flexible setting time and low shrinkage are required for use as repair mortars, tile adhesives, shotcrete, etc. CO_2_-unfriendly CEM I cement or, most recently, the more environmentally friendly calcium sulphoaluminate (CŠA) cement are often used as binders, especially in Asian regions [1,2]. Due to the disadvantages of cementitious systems regarding low adhesion and high brittleness, the addition of polymers to the named mortars is used [3]. Polymer-modified mortars (PMM) show good adhesion, especially on hardened concrete. Nevertheless, at higher temperatures, the main hydration products of Portland cement-based and CŠA-based binders AFt will be decomposed and converted into AFm phases. Consequently, this changes the size and morphology accordingly [4,5]. The existing literature shows that curing temperature will affect the micro-morphology and mechanical properties of PMM [6,7,8]. Likewise, a disadvantage of adding polymers is the temperature dependency [9]. When the ambient temperature exceeds the glass transition temperature of polymers, polymer particles transform from a rigid glassy state to a highly elastic state [10]. These materials can, therefore, lose performance at high temperatures or in the case of fire. Alternatively, the addition of other concrete admixtures, especially carbon nanotubes (CNTs), can help compensate for these disadvantages [11]. CNTs are useful additives for increasing mechanical properties and improving fire resistance. They also have the ability to accelerate hydration, reduce shrinkage [12], and improve durability properties. The high price of CNTs can be negated in the construction chemicals sector. Higher prices are often accepted here for good performance. However, if improperly handled, i.e., poorly distributed, defects can be introduced into the cement matrix, leading to deterioration of its properties. Another disadvantage of CNT addition comparable to PMM is the organic components. Increasingly, there is a demand for single-variety construction to allow for second and/or third life. Construction companies are increasingly trying to establish pure mineral systems (circular economy). It is imperative that the limits for the organic content are complied with.

Alkali-activated binders (hereinafter abbreviated AAM) seem to be an alternative inorganic, resource-saving, and CO_2_-friendly binder regarding a flexible adaptation for construction chemical applications [13]. Ground granulated blast furnace slag (hereinafter abbreviated GGBFS) is the most widely used raw material to produce AAM systems, as its chemical composition and amorphous nature favor the formation of reaction products [14]. For large-scale applications, GGBFS material is only available to a limited extent. Although there are applications, GGBFS is almost fully used, and its availability continues to decline [15,16]. In addition to good durability due to increased acid resistance [17,18] and resistance against sulphate attack [19,20] and chloride penetration [21,22,23], alkaline-activated binders are known to have an insensitive temperature dependence due to non/rarely present AFt phases and improved fire resistance [24]. Consequently, AAM seem predestined for small-scale applications in the construction chemicals industry. For construction chemical applications, however, two essential material properties are mandatory. First, early and controllable solidification is necessary. The literature on immediately solidifying AAM systems can only be found very sporadically, and their advantages are not mentioned further due to an unacceptable processing time [25,26,27]. Second, a low shrinkage behavior is important.

Curing time and shrinkage should be considered as counterparts [28,29,30]. With a reduction of the curing time, a higher shrinkage could often be determined. In particular, high drying shrinkage is considered as a disadvantage [31]. Possible explanations for higher shrinkage are a finer pore structure and lower stiffness of the AAM systems compared to Portland cement-based systems [30,32,33]. Furthermore, the composition of the reaction product has also been identified as a significant factor for shrinkage [34]. A decreasing rate of alkali dosage was said to increase shrinkage due to the enhanced capillary pore pressure and syneresis of C-A-S-H gel; shrinkage occurs without moisture loss during the early autogenous shrinkage of AAM pastes, additionally induced by self-desiccation [35]. According to current state of knowledge, this effect is mainly caused by the spontaneous volume contraction of the silicate gel structure during the silicate polymerization process (also referred to as syneresis) [36,37,38]. To date, the exact shrinkage mechanism of AAM systems is not fully understood. To reduce shrinkage, additives like shrinkage reducers can be used, which, however, reduce the strength. In addition, additives have been shown to have a negative effect in GGBFS-based systems in most cases [29,39] and only have a limited effect in GGBFS/silica fume blended binders [40]. Those GGBFS/silica fume composites are characterized by a low w/b value, which can reduce drying shrinkage.

Both setting time and shrinkage are dependent on the hydration mechanism of AAM. The use of GGBFS with different chemical compositions results in a slight change in phase formation in AAM systems. However, the activator is considered to be the decisive lever for changing the fresh and solid mortar properties of AAM [41,42,43,44]. With increasing solid content in the activator, the differences in mechanical properties due to GGBFS composition decrease [45,46]. Common activators include alkali hydroxides, salts of strong and weak acids, silicates, aluminates, and aluminosilicates [47]. By adding an activator, a gel-like, alumosilicate layer around the individual particles is broken up. Due to the comparatively high CaO content, the C-S-H phases also occur comparable to the hydration of Portland cement [48,49]. In addition, a comparatively high proportion of aluminum is present. The aluminum is usually located at tetrahedral bridging points within the chains and allows the cross-linking between the silicate chains [50,51,52]. This results in the so-called non-cross-linked as well as cross-linked polymers, which promote the formation of C-A-S-H phases [42,47,53,54,55]. C-(N,K)-A-S-H phases are formed due to the addition of alkalis [56]. Hydrotalcite is commonly found in AAM systems in addition to the C-(N,K)-A-S-H phases. In a previous study, it has already been shown that a GGBFS/silica fume blended material that was used in this study produces C-(K)-A-S-H phases and hydrotalcite [57].

For the study of amorphous hydration products, structure-clarifying nuclear magnetic resonance spectroscopy (NMR) is used as the central method [56,57,58,59,60,61,62,63,64]. It allows a quantitative analysis of the cross-linking degree of the C-(N,K)-A-S-H gel. However, NMR sample preparation and measurement method is complex and expensive. Fourier transformation infrared spectroscopy (hereinafter abbreviated FTIR) is an alternative method of analysis for the more inaccurate but simple and rapid estimation/determination of phase formation. FTIR is a simple technique to provide information on distinct effects of the alkali activator solutions on the structure formation in AAM. As a result, FTIR is now being widely used to observe basic stretching and bending vibration bands in AAM systems. In the past, an increasing number of publications on FTIR spectroscopy on AAM systems have been published, and the phase formation has been documented more precisely [65,66,67,68,69]. FTIR is not sufficient to quantify the hydration products of AAM systems [56,67,70]. ^29^Si NMR allows unambiguous quantification of the extent of silicate polymerization in a sample, which is not always possible with FTIR due to problems with band overlap [71]. However, the stretch bonds are optimal for drawing conclusions about the material properties, which are affected by the microstructure. The changes in the bonds affect the flexibility of the structure (e.g., shape changes), linking directly to the gel structure [72]. FTIR analysis can thus be used to obtain information on changes in microstructure inexpensively, much more efficiently, and quickly.

Various methods are used to predict the properties of alkali-activated materials. Multi-factor statistical methods were the choice of various studies in this context. A single-factor method used in other relevant studies cannot reflect the interaction between different factors. Thus, response surface models (RSM) were often chosen to predict and evaluate strength trends [72,73]. In these studies, the prediction models were always based on the activator composition in terms of the modulus of the activator, the choice of activator, and the content of binder. In the correlations, good coefficients of determination of AAM systems between different parameter and composition could be found, which proves that the prediction model has high prediction accuracy and good validity. Subsequently, analytical methods such as FTIR values are mostly used as an evaluation to correlate the properties [74]. From these studies, it can be concluded that FTIR can be directly correlated (without any intermediate step) with the properties of AAM. Moreover, various authors attempted to develop a regression model for shrinkage as a function of composition and explained the shrinkage as a function of the chain lengths of the hydration products and their ion incorporation, which they correlated by FTIR. Good agreement was demonstrated with FTIR [74]. In addition, it was found that more properties from the early reaction process can be described by ultrasound and combined to FTIR results for high accuracy [75]. The aim of this study is to develop a model based on the FTIR method for the efficient prediction of fast-hardening alkali-activated slag/silica fume blended pastes. In this work, it is also shown that further properties of these binders can be directly and accurately estimated from the model. Significantly, the knowledge of the FTIR main peak in the area of 900–1000 cm^−1^ can be used to predict shrinkage and curing behavior depending on the activator used.

## 2. Experimental Section

### 2.1. Starting Material

Three GGBFS with different fineness have been analyzed, namely coarse (cGGBFS), middle (mGGBFS), and fine (fGGBFS). The BET-fineness has been measured according to [76], and the Blaine-fineness according to [77]. The specific surface areas were cGGBFS: 0.6 m^2^/g (BET) and 3965 cm^2^/g (Blaine); mGGBFS: 1.3 m^2^/g (BET) and 7845 cm^2^/g (Blaine); fGGBFS: 2.0 m^2^/g (BET) and 12,105 cm^2^/g (Blaine). The silica fume had a specific surface of 18–22 m^2^/g (measured by BET).

Table 1 presents the chemical composition measured by X-ray fluorescence (XRF) spectroscopy for each of the GGBFS meals and used silica fume. Bulk chemical analysis was conducted using XRF at the Clausthal University of Technology, Institute of Non-Metallic Materials, Germany, in accordance with [78].

**Figure 1:**
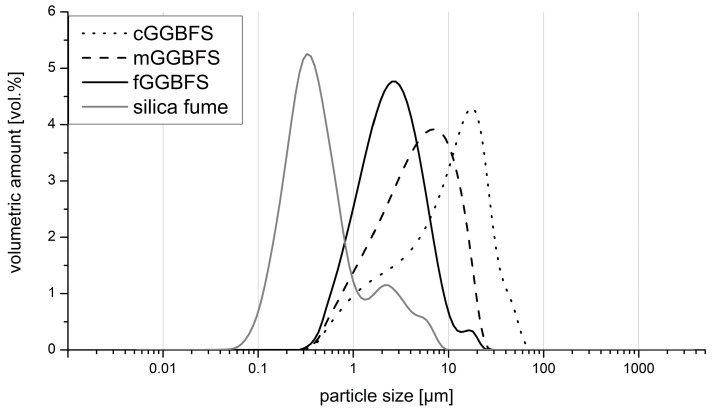
Particle size distribution of the three GGBFS and the silica fume.

Figure 1 shows the particle size distributions of all dry ingredients. The silica fume was the finest component, as expected. The GGBFS were ground to the stated Blaine values.

In this study, the binder was mixed with a comparable low w/b value of 0.3–0.45, depending on the fineness of the GGBFS. It has been shown that the use of silica fume in a system based on GGBFS activated by a combination of alkali-waterglass and alkali-hydroxide improves the rheological behavior while allowing the w/b value to be reduced [80]:

- cGGBFS: w/b-ratio = 0.3
- mGGBFS: w/b-ratio = 0.35
- fGGBFS: w/b-ratio = 0.45

Due to the increase in fineness of the GGBFS, a higher w/b value was necessary for a constant flow spread of the respective AAM mixture. The adjustment of the activator quantity can be carried out in two ways:

1. Constant proportion of K_2_O/slag, thus reducing the proportion of solids in [g/L] in the solution by increasing the water content;
2. Constant percentage of solids in the solution [g/L], thus increasing the proportion of K_2_O/slag.

In this work, variant 2 was chosen because, in this way, a constant pH value of the solution could be guaranteed.

In this work, two water glasses consisting of a potassium silicate solution (K_2_SiO_3_) were mixed with potassium hydroxide (KOH) of different molarities to achieve the highest possible variation of K_2_O and SiO_2_ in the activator solution. The activators used are shown in Table 2. In the following, the water glasses are abbreviated with WG and the potassium hydroxide solution with PH.

### 2.2. Investigated Mix Design by Design of Experiments (DoE)

The binder technology is in search of cost-effective and resource efficient analytical methods to predict the mechanical properties of new binders. Within the scope of the investigations, essential differences and optima should be worked out by varying individual parameters. In empirical experiments, usually only a few of many possible parameters can be varied. Consequently, those designs can be only be used to predict a limited number of material properties.

DoE is a useful statistical approach to increase efficiency by changing several components simultaneously [81]. The main intention is to filter out as much information as possible with as few trials as possible. Due to the economic advantages of DoE, it has already been used in different branches of engineering [81], pharmaceutics [82] and chemistry [83]. In construction material science, DoE is not yet widespread. However, the advantages mentioned above also make DoE interesting and applicable for material sciences [84,85,86]. In this study, DoE was set up using the so-called D-optimal experimental design [87]. To achieve D-optimality, the determinant of the term (X’X)^−1^ in Equation (1) has to be minimized. Consequently, the vector c is maximized:

c = (X’X)^−1^X’n_d_ (1)

The matrix X’X is called the information matrix. That means that by minimizing the negative power, the information is maximized [88].

The mix design was developed by the use of DoE. In this study, the impact of five factors, i.e., WG/PH-ratio, PH type, WG type, the proportion of silica fume, and the GGBFS fineness on the response, i.e., ultrasonic velocity (setting) and FTIR-wavelength (hydration). The ranges and levels of the five factors according to a central composite design (CCD) are given in Table 3. Below 4 wt% of silica fume, the blend was not processable, so a 4 wt% percentage is considered as the lower limit.

### 2.3. Analytical Methods

**Figure 2:**
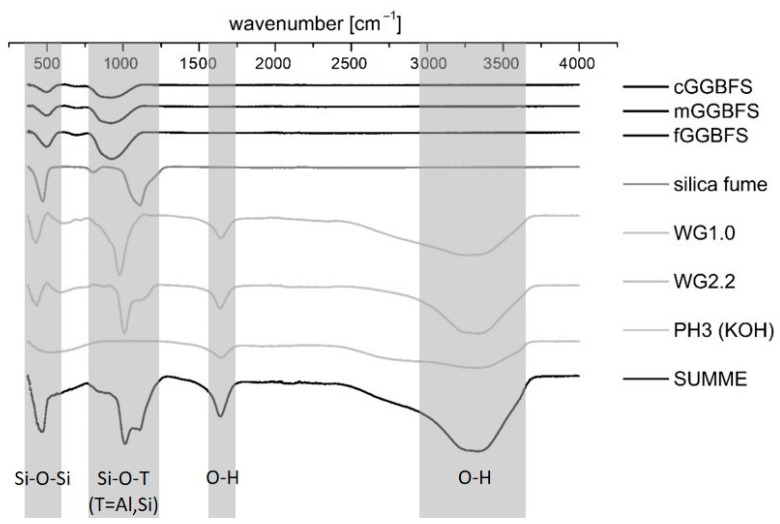
FTIR spectrograms of the raw materials and summed curve at the bottom.

The ultrasonic velocity was monitored in situ with a type of IP-8 ultrasonic measuring system (UltraTest GmbH, Achim, Germany) and the UltraTestLab V2.0 user software. The fresh mortar was mixed with a standard hand mixer. After 60 s of mixing, the material was filled into 130-20 LIV measuring molds equipped with vibration dampers. The chosen apparatus was specially made for in situ measurements of the early setting process. The hydration kinetics was investigated by isothermal conduction calorimetry measurements using a MC-CAL/100P calorimeter (C3 Prozess- und Analysentechnik GmbH, Haar, Germany). For this purpose, all materials were stored for at least 24 h at 20 °C ± 1 °C before preparing pastes by external mixing. For that, 7.5 g of the GGBFS/silica fume binder was weighed. Subsequently, the solution was added with consideration of the solid content of the activator (see Table 3). The material was mixed for 20 s using a spatula to homogenize the mixture and shaken for another 10 s using a “Vortex” mixer before inserting the sample into the calorimeter. The investigations to predict the hydration products were carried out by means of FTIR spectroscopy because the hydrated systems in this work only showed an amorphous hydration peak in XRD (see also [57]). The FTIR–ATR data were obtained using a Bruker ALPHA-Spectrometer (Ettlingen, Germany). The wavenumber range was 500–4000 cm^−1^. The analysis of the spectra for the identification of the functional groups in the hardening AAM system was carried out with the software OPUS. The mixing process was analogous to the previously mentioned isothermal calorimetry. After 24 h, the hardened binder was crushed by agate mortar, and the powder was then placed in the measuring area. Figure 2 shows the spectra of the raw materials. The red curve represents the sum of the given curves. As expected, the GGBFS samples differ only in the peak height and not in the peak location. The potassium water glasses show minor deviations in the maximum peak in the fingerprint region < 1500 cm^−1^, which is 1008 cm^−1^ for WG2.2 and 977 cm^−1^ for WG1.0. The OH bonds of the aqueous solutions are clearly visible at a wavenumber of 1600 cm^−1^ and approx. 3300 cm^−1^. For simplicity, only PH3 has been shown in the diagram as potassium hydroxide (KOH). In the course of the studies, only the strain vibrations of Si-O-T units were observed. These are characteristic of the formation of the reaction products. As stated in the literature part, in Portland cement and AAM systems, the Si-O-T stretch belts of the C-(A)-S-H phases can be found at a wavenumber in the range of 900–1000 cm^−1^ [65,66,67,68,69,89,90].

A Quanta FEG 250 environmental scanning electron microscope (ESEM; FEI, Hillsboro, OR, USA) was used for the scanning electron examinations. For the microscopic examinations, sections of the samples to be analyzed were prepared and carried out in low-vacuum mode using water vapor as ambient gas. To check the shrinkage deformation, shrinkage prisms were examined in accordance with [91]. Contrary to the dimensions in the DIN standard, smaller shrinkage prisms with dimensions of 2 × 2 × 11 cm^3^ were produced for the tests to handle the faster setting time. Most of the shrinkage tests performed on AAM were carried out by producing and measuring shrinkage prisms with higher dimensions. Therefore, the samples were stored in the formwork for a defined period of time (usually 24 h) before they were demolded, and a zero value was determined [29,33,92]. During the first 24 h of hydration, however, the major change in shape already occurs. Consequently, in this study, samples have been deformed and measured for zero value directly after hardened. Shrinkage cracks start and occur in the hardened state when no more deformations can be absorbed. Finally, the investigations to measure the pore sizes were carried out in accordance with [93] with a Poremaster from Quantachrome (Boynton Beach, FL, USA). The samples were measured after 24 h in fragments up to 5 mm.

## 3. Results and Discussion

The following approach was used in this study:

(i) Determination of setting time and FTIR wavenumber after 24 h of hydration using DoE;
(ii) Definition of significant factors by DoE;
(iii) Correlation of FTIR wavenumber with hydration kinetics and shrinkage behavior;
(iv) Development of a predictive model;
(v) Evaluation of the predictive model.

### 3.1. DoE Results and Statistical Properties of the Investigated Mixtures

#### 3.1.1. Determination of the Minimum Number of Test Points by DoE

**Figure 3:**
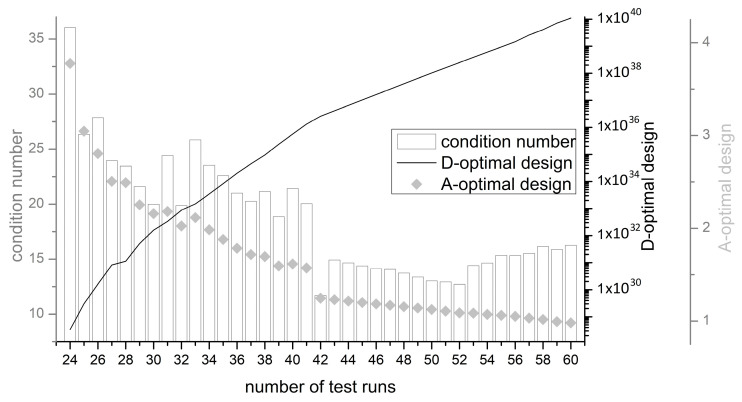
D- and A-Optimality values as well as the condition number in dependency of the number of test runs.

In the run-up to the experimental design, a centrally composed response surface design was chosen in order to analyze the interactions between the experimental parameters. No additional randomization of the runs was performed to ensure reproducibility of the DoE plan. Consequently, only the randomization automatically generated by Minitab^®^ version 18 (the assignment of the experimental unit is not subject to any known random mechanism) was used. In order to obtain the maximum information with the smallest possible number of trials, the experimental runs were kept to a minimum by D-optimization. The original experimental design was created with the sequential method and improved with the Fedorov method. In this way, more possible experimental designs can be considered. Although the generation of an initial experimental plan takes more process time on the one hand, on the other hand, higher D-optima are set for the same number of points [52]. Figure 3 shows the calculated D-optima for different number of trial points, starting with 24 trial runs. From 42 points on the *x*-axis, a bend in the line for the D-optimality can be seen. At the same time, the mean variance of the regression coefficients (A-optimality) also decreases sharply from a number of 42 trials. Consequently, 42 trial points were selected for the study. This experimental arrangement is selected according to the criteria of D-optimality and, at the same time, A-optimality. With a higher number of experimental runs, the respective optimality increases, of course, but leads to an enormous experimental effort. As a third characteristic, the condition number can also be used, which provides information about the collinearity of the experimental design. A low collinearity is advantageous for the interpretation and accuracy of the results, as there are fewer correlations. The detailed overview of 42 test points is given in Appendix A.

#### 3.1.2. Curing Properties Measured by Ultrasonic Test

In preliminary investigations, it was found that the start and end of curing of alkali-activated GGBFS/silica fume blends can be determined very precisely by means of ultrasonic measurement that correlates to the Vicat test [46].

**Figure 4:**
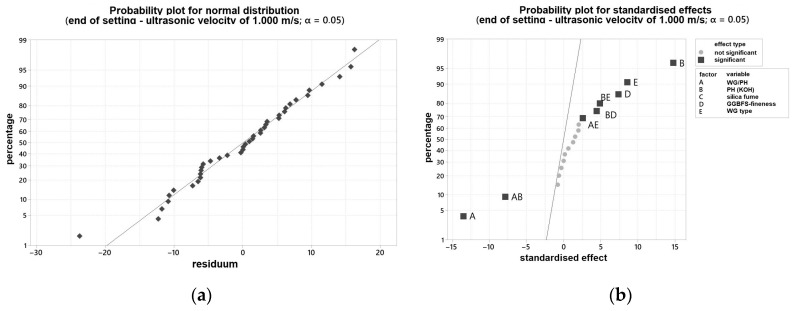
Probability plot for: (**a**) normal distribution for the end of setting measured by ultrasonic test; (**b**) standardized effects for the end of setting measured by ultrasonic test.

In order to be able to make a statement about the influence of various varied parameters on the curing behavior, the results of the ultrasonic test were evaluated with the statistical software Minitab^®^. Figure 4a shows the probability plot for normal distribution, and Figure 4b the probability plot for standardized effects for the time point mentioned. All individual values are listed in Appendix A. The two-sided confidence level was set at 95% for all intervals. The time at which the ultrasonic velocity through the blends is 1000 m/s was determined. This time also correlated with the end of setting of the blends.

Two samples showed a gel-like structure and an ultrasonic velocity of <500 m/s even after 24 h and were, therefore, not considered further. Both samples have been activated solely with the WG2.2. Otherwise, an evaluation would not be possible and would lead to false results with a low R^2^. With the modification, an R^2^ of 97.73% can be set.

**Figure 5:**
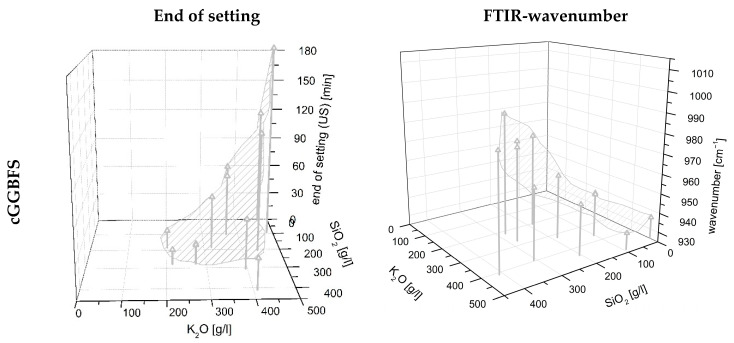

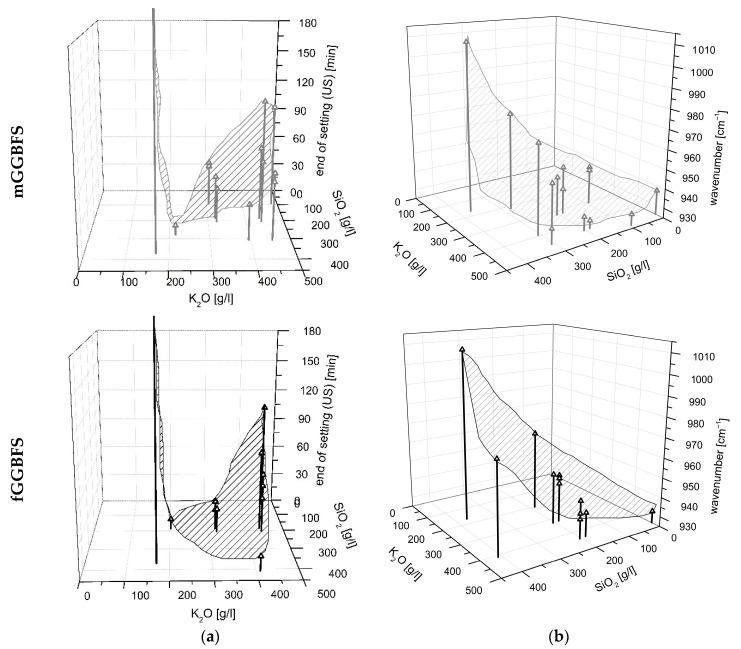
Surface model to determine (**a**) end of setting and (**b**) FTIR wavenumber in dependency of the activator composition by using cGGBFS (**top**), mGGBFS (**middle**), and fGGBFS (**bottom**) as raw materials.

Therefore, the curing behavior is significantly dependent on the used PH type, the WG type, as well as their combination. That means that the K_2_O/SiO_2_ ratio and the respective amount are the major levers to adjust curing behavior. In addition, the interactions of the factors mentioned must be considered significant. Furthermore, the grinding fineness of the GGBFS is important for the curing time, but to a minor extent. In Figure 5a, it can be seen that the end of setting (measured by ultrasonic test (US)) decreases with increasing SiO_2_ content, thus increasing silica modulus. Some of the samples solidified after less than 10 min. In the case of a too high silica modulus, the samples did not solidify. As expected, increasing the fineness of the GGBFS leads to an acceleration of the reaction (linear influence).

#### 3.1.3. Hydration Properties Measured by ATR-FTIR

According to the existing publications, the C-(N,K)-A-S-H phases of AAM are found in the band region of 900–1000 cm^−1^ and are characterized by the Si-O-T (T = Al, Si) asymmetrical stretching vibrations. In various sources, the formation of the chain length is also characterized in more detail. Thus, Q2 units are found in the 960 cm^−1^ range. Q1 short-chain units are defined in the range around 850 cm^−1^ [89,90]. An increasing band number in this wave range indicates a long-chain C-(N,K)-A-S-H phase and, thus, a high degree of cross-linking. According to Ravikumar and Neithalath [68], higher wavenumbers are an indication of the formation of Q3 silicate units. Those are primarily formed by a low Ca/Si ratio in the C-(N,K)-A-S-H phases and link the existing silicate chains. As a result, a higher degree of polymerization is achieved.

The incorporation of Al further reduces the wavenumber of FTIR in the area 900–1000 cm^−1^. According to various publications [53,89], the incorporation of aluminum into the C-S-H phases modifies the chemistry of the Si-O-T bonds with a subsequent reduction of the wavenumber. At the same time, Si-O-Al bonds appear in the 875 cm^−1^ range, indicating tetrahedral-bonded aluminum. Under more alkaline activation conditions, the asymmetric stretching mode of Si-O-T bonds is observed at a lower wavenumber (980 cm^−1^). As a result, a decreased silica modulus in the activator leads to a reaction product with a higher Ca/Si ratio. That favors depolymerization by breaking the Si-O-Si bonds to create Si-O groups and shorter chain lengths [27,94]. Nevertheless, it should be noted that most studies are conducted on samples that were stored for not less than seven days. An increase in wavenumber by reaction time could be detected by different authors [95,96].

**Figure 6:**
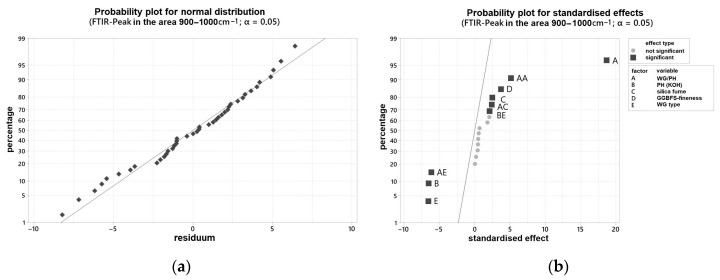
Probability plot for: (**a**) normal distribution for the FTIR peak in the area 900–1000 cm^−1^; (**b**) standardized effects for the FTIR peak in the area 900–1000 cm^−1^.

The shifted peak positions in the range 900–1000 cm^−1^ measured by FTIR were determined after 24 h of curing for each sample and evaluated with the software Minitab^®^ (individual values see Appendix A). The two-sided confidence level was set at 95% for the analysis of the FTIR peaks for all intervals. The R^2^ of the examined variables is 97.36%, so that a very accurate prediction of the FTIR peak in the range 900–1000 cm^−1^ can be assumed depending on the examined variables. Figure 6a,b show the probability network for normal distribution and the probability network for standardized effects. According to this, many factors have an influence on the position of the FTIR maximum peak. It is obvious that the FTIR shift depends almost exclusively on the composition of the activator. In particular, the activator composition is of decisive importance and, at the same time, shows a quadratic influence (AA). The grinding fineness of the GGBFS also has an influence on the peak development, albeit to a lesser extent. The marker, depending on the silica fume content, is almost on the reference line, the level of 0.05, and can still be considered statistically significant. Since the point C (silica fume content) is displayed to the right of the dividing line, it can be concluded that with an increase in the factor level (increase in silica fume content), the wavenumber of the FTIR maximum peak also increases slightly.

Since the activator has the most significant influence on the position of the FTIR peak, Figure 5b shows the wavenumbers as a function of the amount of K_2_O and SiO_2_ in the activator. The shift of the peak in the range 900–1000 cm^−1^ to a smaller wavenumber with increasing K_2_O content in the activator is evident. In addition, it can be seen that for the samples using finer slags, mGGBFS and fGGBFS, the wavenumber of the respective maximum peak is, on average, at a lower level compared to the peaks for the blends using cGGBFS. The measured IR spectrum of AAM systems always represents a total of unhydrated GGBFS and the reaction products. For the analysis of the hydration process, the peak position and the shift over time are of particular interest.

In Figure 5, a shifted perspective compared to the figures for end of setting was chosen for representational reasons. However, it is noticeable that the inlaid area is exactly inversely proportional to the determination of the end of setting in Figure 5a. Consequently, the hypothesis can be made that the phase development measured by FTIR and curing time correlate and enable modelling.

### 3.2. Detailed Investigations

The DoE studies have shown that the setting and hydration mechanism of GGBFS/silica fume blends depend significantly on the choice of activator. The development of the FTIR peak is significantly influenced by the activator composition. It is known that the main FTIR peak consists of the overlap of different peaks [57]. The correct analysis of the deconvolution of the peaks and the comparative analysis depends on them. However, the location of the main FTIR peak allows an estimation of the cross-linking of the silica gel, which, in turn, has been shown to determine the properties of the hardening material.

#### 3.2.1. Sample Overview

**Figure 7:**
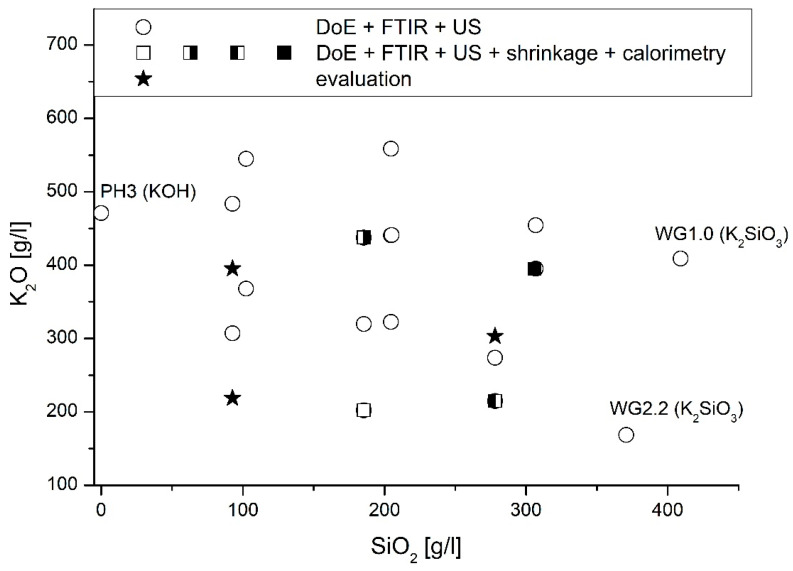
Sample overview.

To analyze the influence of the activator composition on the microstructural properties in more detail, characteristic samples were analyzed. Figure 7 gives an overview of test points.

Four samples, marked by (half-)filled squares in Figure 7, were examined in more detail in the following. Three samples, indicated by stars in Figure 7, were subsequently investigated to evaluate the developed prediction model. Table 4 gives a detailed overview of the samples.

For the detailed investigations, however, several components had to be kept constant. From the DoE results, it can be concluded that the activator composition significantly determines the binding properties. Furthermore, there is a quadratic effect. The fineness of the GGBFS and the silica fume content are less significant and can be evaluated as a linear influence. This agrees with different literature statements that the influence of GGBFS decreases with increasing alkali content in the activator [45,46].

Hence, the Centre Point values (see Table 3) were set as fixed, as raw material mGGBFS was used, and the silica fume content was set to 8 wt%.

#### 3.2.2. Hydration Kinetics

**Figure 8:**
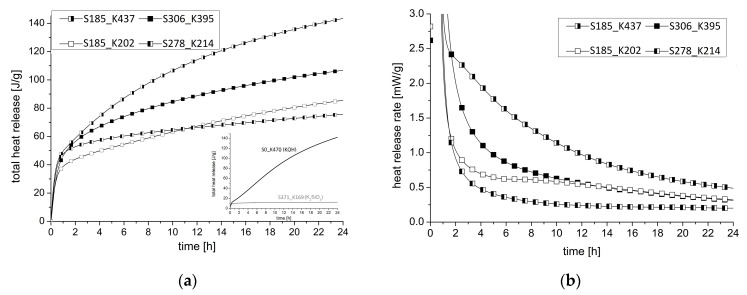
(**a**) Total heat release of selected samples; (**b**) Heat release rate of selected samples.

The heat of hydration in a period of 24 h is shown in Figure 8. Figure 8a shows in the below right the hydration curves for blends activated exclusively with a SiO_2_-rich activator WG2.2 (S371_K169) and exclusively with a K_2_O activator PH3 (S0_K470). It is noticeable that the use of WG2.2 is immediately followed by a plateau after a small initial increase of heat release. The material was still not solid after 24 h. When PH2 is chosen as activator, a steady, almost linear increase over 24 h can be seen. By combining the two solutions, a clear acceleration of the hydration and a clear increase of the initial period in the first 10 min of hydration can be measured. The samples with a comparatively high potassium hydroxide solution as activator show a clear increase even after approx. 1 h. It is obvious that a high SiO_2_ content in the activator weakens the post-hardening.

When analyzing the heat release rate of the four samples in Figure 8b, it is noticeable that a high SiO_2_ content in the activator leads to a rapid decrease in the heat of hydration during the induction period within 2 h. This process correlates with the end of setting measured by ultrasonic testing. With a higher K_2_O content in the activator, for example, samples S185_K437 and S306_K395, there is a gradual decrease in the heat of hydration and the material remains flexible at the end of the solidification period and is thus buffered. Subsequently, an acceleration period takes place only in the samples with a low SiO_2_ content, which is strongly buffered in sample S185_K437.

According to Fu et al. [97], different processes can be observed based on the heat release rate:

In the initial period, there is the hydrolysis of alkali activator dissolution of Ca^2+^ and Si^2+^. Following this, a dissolution of slag will appear in the induction period [98] before C-A-S-H and hydrotalcite are formed (acceleration period). According to this, a gradual dissolution and reaction formation occurs in sample S185_K437. According to the authors, the degree of hydration can be estimated. In a previous study, the degree of hydration of comparable GGBFS/silica fume samples was investigated [57]. The results in this study can confirm that the degree of hydration increases with a high K_2_O amount in the activator that correlates with the heat release measurements.

The correlation of the activator composition on the hydration of the GGBFS/slag mix is shown schematically in Table 5. Accordingly, an activator with a high K_2_O and low SiO_2_ content should be selected. The silica modulus is, therefore, important for the acceleration period but is not the decisive criteria for the hydration process in the initial period. The activator composition is a complex system of alkalis and SiO_2_ amount.

However, two points should be considered:

- The combination of a high K_2_O content with a low SiO_2_ content cannot be increased arbitrarily. By using an activator with a modulus < 0.5, the heat of hydration decreases and leads to a negative effect (see small diagram in Figure 8a).
- If too much SiO_2_ is added compared to K_2_O, resulting in a modulus > 2.0, the sample approaches the effect in Figure 8a for WG2.2 and no solidification occurs after 24 h.

#### 3.2.3. Time-Dependent Deformation of Mortars

The following microstructural investigations were carried out on mortars. The used mixture in this study is based on the structural density-optimized Ultra High Performance Concrete (UHPC) formulation (M3Q) tested extensively in the priority programme (SPP) 1182 funded by German Research Foundation (DFG) [99].

Of the four samples tested by calorimeter, the shrinkage deformation was determined over 14 days. In particular, the deformation was observed after 24 h in more detail, during which approx. 80% of the total shrinkage deformation has already taken place. The sample that was only excited by PH3/KOH (S0_K470) was also examined. AAM systems have a high shrinkage value compared to Portland cementitious binders. As described, the shrinkage was investigated in this work using small test prisms. The end of curing (1000 m/s) measured by ultrasonic testing was used as the zero value and start time of the measurement. At this point, the samples could be demolded without any problems. After the binder has solidified, changes in shape can lead to forced stresses and thus cause cracks in the binder matrix. The zero value of the samples was determined immediately after the end of solidification, as described in the analytical methods section.

**Figure 9:**
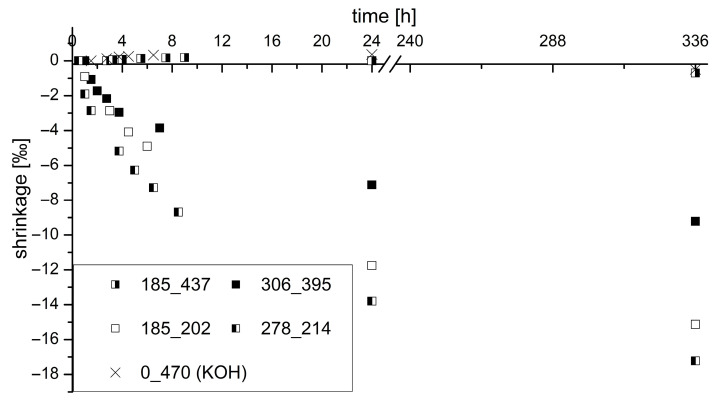
Shrinkage of investigated samples over a period of 14 days with focus on 24 h.

Figure 9 shows the shrinkage values for the tested samples over the stated period. A direct correlation between the shrinkage rate and the potassium and silicon content in the activator can be observed. The modulus (SiO_2_/K_2_O ratio) is not predominantly significant. The amount of potassium in the activator significantly determines the degree of shrinkage in the binder system. A high potassium content reduces shrinkage, for example, 0‰ at 437 g/L K_2_O compared to −9.9‰ at 202 g/L K_2_O and the same silicon content in the activator. The higher SiO_2_ content also leads to a slight increase in shrinkage of the GGBFS/silica fume blend (sample S278_K214).

In addition, a slight increase in volume of 0.36‰ after 24 h can even be seen in the sample without SiO_2_ amount in the activator (S0_K470). This behavior was also observed by Melo Neto et al. [92] in their investigations. The expansive behavior is attributed to autogenous shrinkage. According to this, there is a reabsorption of free water in the mixture in the early stage before the start of the acceleration phase in the system. The water demand increases due to an incipient C-(K)-A-S-H phase formation. According to the authors’ assumption, the free (bleeding) water is transported to the places where self-drying is more intensive.

However, when looking at the results, the mentioned expansions can only be noticed sporadically. Obviously, a high K_2_O content is responsible for the very rapid formation of free water, which leads to the named expansion at an early hydration age. At the same time, this expansion of the autogenous shrinkage acts as a shrinkage reducer, since the samples with high K_2_O content have low shrinkage after 24 h.

**Figure 10:**
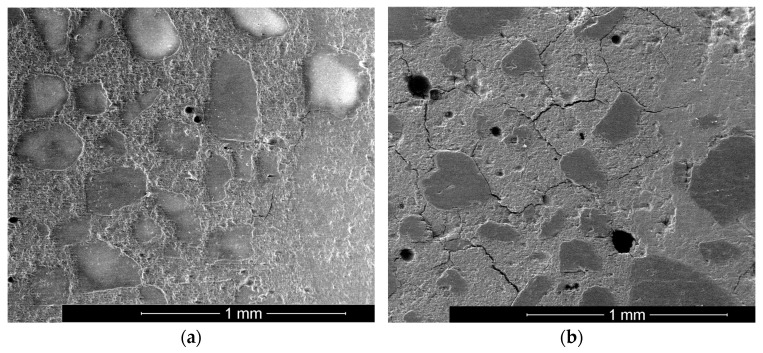
Characteristic ESEM picture of samples S185_K437 (**a**) and S278_K214 (**b**).

Form changes can be the exclusion criterion for being able to use materials for building chemical applications. The bonding behavior was tested to analyze the influence of deformation due to shrinkage. A standard concrete C50/60 was produced. A hole was drilled, and the investigated mortars filled in. After 24 h of curing, sections were made, and the bond area analyzed by ESEM (see Figure 10a,b).

Samples with high shrinkage (S278_K214) and low shrinkage (S185_K437) were tested. In the case of high shrinkage, cracking of the system occurred in each case. The cracks mainly proceed in the fresh mortar (left part in each image). At first, the bonding area looks stable for both samples. Nevertheless, for sample S278_K214, some cracks reach into the transition zone and reduce the adhesion behavior of GGBFS/silica fume blends.

The results regarding the shrinkage behavior are of particular interest in the case of highly alkaline AAM systems, as it could be shown that neither superplasticizers nor commercially available shrinkage reducers are currently effective in low w/b-ratio systems consisting of GGBFS modified with silica fume [40].

## 4. Modelling and Evaluation

### 4.1. Modelling of Curing and Hydration Properties of AAM Systems

In the equations for determining the FTIR value, it is noticeable that there are many interactions in addition to quadratic effects (see probability network in Figure 6b). At first glance, it seems that a system cannot be represented as a function of one variable (x-value). A three-dimensional model has to be developed. Since the grinding fineness of the GGBFS and silica fume content in the binder system shows almost linear effects, the values can be changed after modelling by multiplication/addition in accordance with DoE software Minitab^®^:

- From the determined FTIR results of the Si-O-T peak that is decisive in predicting the performance properties, the wavenumber in the range 900–1000 cm^−1^ can be predicted. As mentioned before, a high wavenumber stands for a high number of cross-links of the silicate units. Figure 5 shows that the effective areas by using GGBFS with fineness values of 8000 cm^2^/g and 12,000 cm^2^/g differ only slightly but show a shift towards lower wavenumbers.
- With increasing silica fume content (decreasing content of GGBFS in the system), more amorphous SiO_2_ is available, and slightly more cross-linked C-(K)-A-S-H chains are formed in the system.

In the model development, the values are to be created as a function of the activator concentration of K_2_O and SiO_2_ in [g/L]. From the surface models in Figure 5, it can be seen that the values take a parabolic course and have an almost funnel-shaped convergence. As a three-dimensional function, a quadric in the three-dimensional space R3 known from mathematics has to be used.

Characteristic edge range points are given for the mGGBFS:

➢ g(SiO_2_;K_2_O) = FTIR value of Si-O bond 900–1000 cm^−1^
➢ g(350;375)  = 965
➢ g(0;475)   = 930
➢ g(200;250)  = 935.

By substituting the three points into the Equation (2) for a midpoint quadric:

(2) $$ax_{1}^{2}+bx_{2}^{2}+cx_{3}^{2}=1$$

the following parameters are obtained:

$$a=-\frac{39,643}{87,542,587,500}\approx -4.53\times 10^{-7}\;b=\frac{16,639}{80,247,371,875}\approx 2.07\times 10^{-7}\;c=-\frac{10,613}{9,629,684,625}\approx -1.10\times 10^{-6}$$

Consequently, a function $g\left(x_{SiO_{2}};y_{K_{2}O}\right)$ can be determined by including the K_2_O and SiO_2_ content to determine the FTIR peak value, Equation (3).

(3) $$g\left(x_{SiO_{2}};y_{K_{2}O}\right)=\sqrt{\frac{-4.53\times 10^{-7}{x_{SiO_{2}}}^{2}+2.07\times 10^{-7}{y_{K_{2}O}}^{2}-1}{-1.10\times 10^{-6}}}$$

Finally, pre-factors and summands have to be determined in order to consider the different degrees of grinding fineness of the GGBFS (*z_Blaine,GGBFS_*) and the small but influential silica fume content (*z_silica_*), Equation (4). According to DoE results, fractionally rational function is suitable for the prefactor of the grinding fineness of the GGBFS. Especially for the cGGBFS, the FTIR wavenumbers are to be evaluated significantly higher. The following formula applies to GGBFS with grinding fineness *z_Blaine,GGBFS_* ≥ 4000 cm^2^/g. An exponential function is used to take the silica fume content into account.

(4) $$g\left(x_{SiO_{2}};y_{K_{2}O};z_{Blaine,\;GGBFS},\;z_{silica}\right)=\;\left(1+\frac{1}{\left(z_{Blaine,GGBFS}-3900\right)}\right)\sqrt{\frac{-4.53\times 10^{-7}x_{SiO_{2}}^{2}+2.07\times 10^{-7}y_{K_{2}O}^{2}-1}{-1.10\times 10^{-6}}}+\left(1.3^{z_{silica}-8}-1\right)$$

**Figure 11:**
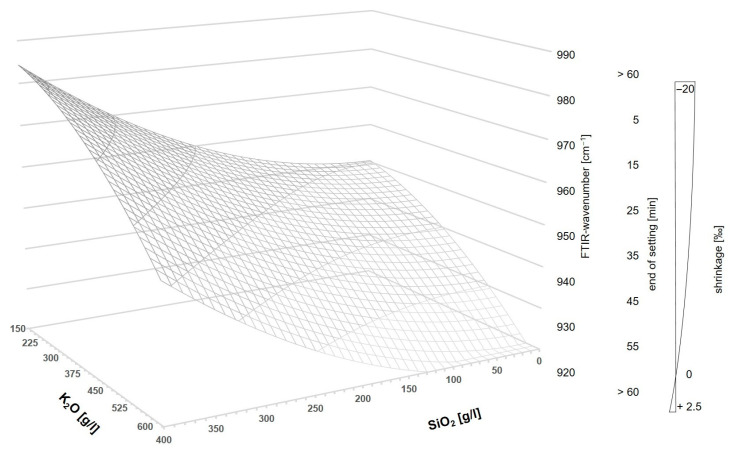
Three-dimensional model to predict AAM properties in dependency of the activator composition.

Looking at Equation (4), it is noticeable that the structure of the equation corresponds to that of a single-shell hyperboloid. By limiting the *x* and *y* values for exclusively positive values (SiO_2_ and K_2_O), the following prediction model in Figure 11 results in the three-dimensional coordinate system for AAM systems. The model applies to mixtures using GGBFS with a grinding fineness of 8000 cm^2^/g and shifts slightly to higher wavenumbers when a coarse GGBFS is chosen.

Based on the correlations analyzed in this study, further factors can be taken from the model, and the properties can be estimated on the basis of the activator composition. As shown beforehand, the activator composition can also be used to infer other factors of the binder system, such as the end of setting (inversely proportional to FTIR) and the shrinkage behavior.

Nevertheless, it should be noted that the above model should be used for a SiO_2_ content ≠ 0, and the user is advised not to use a pure activator system based on an alkali hydroxide solution as this will lead to a retarded hydration process (see calorimetry result in Figure 8).

The aim should be to adjust the sample so that the wavenumber after 24 h is in the range 930–940 cm^−1^. In this way, a faster curing time <60 min, a good bonding behavior due to a shrinkage value near to 0‰, and a sufficient hydration process can be ensured.

### 4.2. Evaluation of the Model Robustness

**Figure 12:**
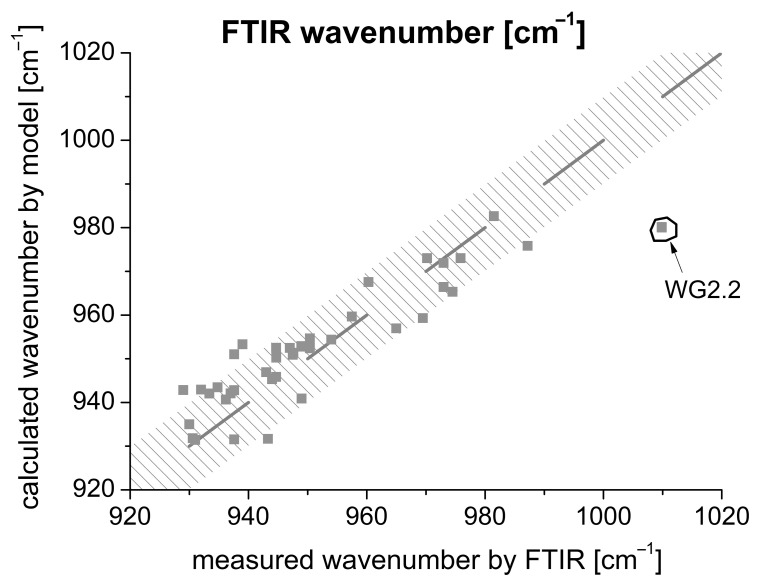
Comparison between model and measured FTIR values.

The evaluation of the developed AAM model was twofold:

On the one hand, the robustness was checked. Figure 12 shows that there are only minor deviations between the model and measured value. The dashed area in Figure 12 represents a deviation of 10 cm^−1^ for the FTIR wavenumber. Inserting the determined 42 values results in a deviation of the FTIR wavenumber in the range ±10 cm^−1^. Consequently, a very good approximation can be assumed. Only the use of an activator consisting exclusively of the water glass type WG2.2 results in stronger deviations. As already mentioned, the material is still gel-like after 24 h of hydration and has not solidified.

On the other hand, FTIR, setting time, and shrinkage measurements were carried out on three randomly selected samples (located in the interstices; see stars in Figure 7) to exclude an influence of the activator type on the one hand and to analyze a possible crack formation on the other hand.

From the K_2_O and SiO_2_ data of the activator solution, an FTIR value can be determined by means of Equation (4). The FTIR values could be adjusted very accurately.

FTIR wavenumber:

- g(278;303;8000;8) = 960.4 → measured FTIR wavenumber: 964
- g(093;219;8000;8) = 950.2 → measured FTIR wavenumber: 949
- g(093;395;8000;8) = 939.4 → measured FTIR wavenumber: 944.

Setting time (US) and shrinkage value:

- g(278;303;8000;8): FTIR = 960.6 → 24 min → −10.76 [‰]
- g(093;219;8000;8): FTIR = 950.2 → 32 min → −8.86 [‰]
- g(093;395;8000;8): FTIR = 939.2 → 50 min → −2.89 [‰]

In Figure 12, the measurements are compared with the estimated values. It can be summarized that the measurements are in the required range with minor deviations. The largest deviation of 5 min (50 min to ≈45 min) is present in sample g(093;395;8000;8). Since the model only gives an approximation, a very good agreement can be assumed.

### 4.3. Discussion

The FTIR method has limitations for evaluating the hydration process of AAM. Characterization and interpretation of the results is difficult as the Si-O-T (T = Al, Si) vibrations from the hydration product of AAM and the unreacted slag and silica fume yield an overlapping spectrum [95]. Nuclear magnetic resonance spectroscopy (NMR) is required for the precise characterization of the hydration products. Although FTIR does not allow detailed structural analysis, it provides insight into the nanostructure and, therefore, the polymerization degree. Conclusions can be drawn about the hydration process with only little effort. FTIR is a cost-saving analysis method, especially against the background of resource efficiency during the examination. The fast evaluation saves costs in laboratory operation and enables simple and efficient work.

The calorimetry experiments provide further insight into the hydration kinetics and are related to the FTIR wavenumbers. The formation of cross-linked or non-cross-linked hydration products is confirmed by the heat released during alkali activation. As reported in previous studies, the total heat release of AAM is less than that of OPC [100,101]. As mentioned in [102], a higher amount of alkali leads to a higher heat release. This, in turn, leads to a shorter chain length, as shown in the studies. The shorter chain length, in turn, can be easily determined by an FTIR measurement.

**Figure 13:**
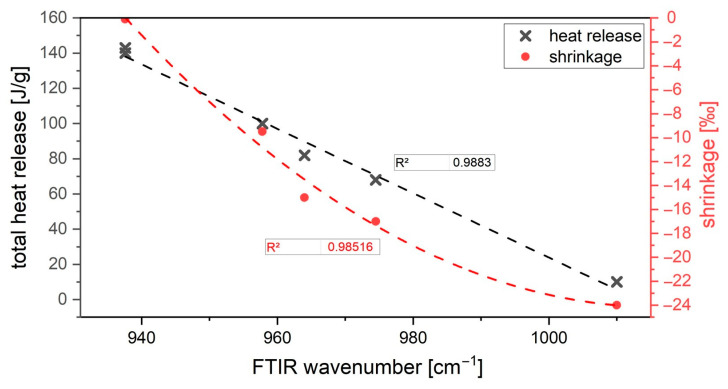
Correlation of FTIR wavenumber, calorimetry results, and shrinkage behavior.

All properties were found to depend significantly on the composition of the activator. The properties are influenced differently by the activator components K_2_O and SiO_2_. It could be shown that the silica modulus (MS ratio), which is regarded as the decisive lever in many studies, cannot be regarded as the sole lever for the fresh and hardened concrete properties. The influence of the alkali component K_2_O compared to SiO_2_ is more significant. The activator dosage exerts a quadratic and not a MS-related conditioned linear influence on the performance properties (see Figure 13). It cannot be defined that an increasing amount of activator leads to a stronger shrinkage. The shrinkage correlates primarily with the K_2_O content in the activator solution. This is in accordance with the calorimetry results. The higher shrinkage due to activation of AAM systems with a low K_2_O content in the binder system is a consequence of the formation of silica gel during hydration. The silica gel with many cross-links forms a lot of water during hydration and subsequently dries out. As a result, micro cracks appear in the material. A high K_2_O content blocks the formation of strongly cross-linked silica gels within the C-(K)-A-S-H phases and thus, at the same time, ensures a shorter chain length (measured by FTIR). The effects are slightly enhanced by the amount of SiO_2_ in the activator. The usual tests, in which the test specimen remains in the formwork for 24 h, are unsuitable because the initial deformation is not considered.

## 5. Conclusions

This study shows that the fresh and setting properties of cementitious GGBFS/silica fume mixtures can be predicted precisely. DoE was shown to be an effective tool for modelling properties of AAM-based binders. Only small amounts of material are sufficient to determine the FTIR wavenumber, which correlates with the setting time and shrinkage behavior. The results of the FTIR measurements are strongly influenced by the atomic environment. Although no exact estimation of the degree of hydration is possible, an estimation of the hydration phase formation can be made. The FTIR wavenumbers have been shown to allow correlations to cross-linking of silica gel. The study shows that these finally allow a pinpoint estimation of the shape properties.

With the present methodology, the mechanical properties of a GGBFS/silica fume blend can be predicted. The results of this study can be applied to all alkali-activated slag systems. The equation is applicable for latent hydraulically-reacting raw materials. If the chemical composition of the GBFS differs significantly between slag batches, the modelling equation may need to be adjusted by a prefactor. In that case, a prefactor for the chemical composition of the activator (Na or K) and the chemical composition of the GBFS could be developed in further research. Furthermore, the general methodology in this study can be applied to other precursor materials, since FTIR analysis allows conclusions to be drawn about the structure of AAM systems as shown.

FTIR measurement can be used as a quick and material-saving verification method. Finally, it is possible to create fast-setting and dimensionally stable binders for the building chemistry sector.

## Figures and Tables

**Table 1:** Chemical composition of the investigated GGBFS and silica fume Silicoll P from Sika AG.

wt%	SiO_2_	Al_2_O_3_	Fe_2_O_3_	CaO	MgO	SO_3_	Na_2_O	K_2_O	Cl	Mn_2_O_3_	S	LOI *
cGGBFS	38.10	9.89	0.41	40.33	5.68	2.74	0.41	0.74	0.01	0.58	1.12	
mGGBFS	38.36	9.94	0.40	39.95	5.74	2.72	0.41	0.76	0.01	0.58	1.13	
fGGBFS	38.51	10.02	0.41	39.68	5.79	2.74	0.42	0.75	0.01	0.57	1.10	
silica fume	96.57	0.06	0.06	0.51	0.25	<0.04	0.16	0.73		0.02		1.41

* Loss on ignition: 1025 °C (acc. [79]).

**Table 2:** Chemical composition of the activator used in this study.

	WG2.2	WG1.0	PH1	PH2	PH3	PH4	PH5
K_2_O [g/L]	169	409	235	353	471	589	706
SiO_2_ [g/L]	371	409	-	-	-	-	-
Solid content [%]	35	45	23	32	40	47	54
Modulus [-]	2.2	1.0	-	-	-	-	-

**Table 3:** Overview of the variations investigated for the AAM systems.

Factor	Type	Bottom Star Point	Lower Limit	Centre Point	Higher Limit	Upper Star Point
WG/PH	steady	0/100	25/75	50/50	75/25	100/0
PH (K_2_O)	steady	PH1	PH2	PH3	PH4	PH5
Silica fume	steady	4 wt%	6 wt%	8 wt%	10 wt%	12 wt%
GGBFS-fineness	categorical		cGGBFS	mGGBFS	fGGBFS	
WG type	categorical		WG2.2		WG1.0	

**Table 4:** Sample overview: four samples analyzed in detail, and three samples evaluated.

Factor	WG/PH	PH (K_2_O)	Silica Fume	GGBFS Fineness	WG Type	SiO_2_ [g/L]	K_2_O [g/L]
S185_K437	50/50	PH5	8 wt%	mGGBFS	WG2.2	185	437
S185_K202	50/50	PH1			WG2.2	185	202
S278_K214	75/25	PH2			WG2.2	278	214
S306_K395	75/25	PH2			WG1.0	306	395
S278_K303	75/25	PH5	8 wt%	mGGBFS	WG2.2	278	303
S093_K219	25/75	PH1			WG2.2	093	219
S093_K395	25/75	PH3			WG2.2	093	395

**Table 5:** Correlation between activator composition and hydration process (↑ = high heat of hydration; ↓ = low heat of hydration).

SiO_2_	K_2_O	Initial Period	Induction Period	Acceleration Period
high	high	↑	↑	↓
high	low	↑	↓	↓
low	high	↑	↑	↑
low	low	↓	↓	↑

## Appendix A

**Table A1:** Detailed overview of tested specimen including data composition.

DoE nr.	WG/PH	PH (K_2_O)	Silica Fume	GGBFS Fineness	WG Type	End of Setting [min]	FTIR Wavenumber [cm^−1^]
1	0/100	PH3	8 wt%	fGGBFS	WG1.0	101.5	931.0
2	50/50	PH1	8 wt%	fGGBFS	WG2.2	9.5	965.0
3	100/0	PH3	8 wt%	fGGBFS	WG1.0	13.5	970.2
4	50/50	PH5	8 wt%	fGGBFS	WG2.2	75.0	937.6
5	50/50	PH3	4 wt%	fGGBFS	WG1.0	33.0	937.6
6	50/50	PH3	12 wt%	fGGBFS	WG1.0	56.5	944.7
7	50/50	PH1	8 wt%	fGGBFS	WG1.0	22.5	950.4
8	50/50	PH5	8 wt%	cGGBFS	WG1.0	225.5	949.0
9	50/50	PH1	8 wt%	cGGBFS	WG2.2	17.5	973.0
10	0/100	PH3	8 wt%	mGGBFS	WG1.0	97.5	937.6
11	100/0	PH3	8 wt%	mGGBFS	WG2.2	-	1009.9
12	100/0	PH3	8 wt%	cGGBFS	WG1.0	28.5	981.5
13	0/100	PH3	8 wt%	cGGBFS	WG2.2	116.0	936.2
14	50/50	PH3	4 wt%	mGGBFS	WG1.0	59.0	932.0
15	50/50	PH3	4 wt%	cGGBFS	WG2.2	51.0	957.5
16	75/25	PH4	10 wt%	mGGBFS	WG1.0	62.5	958.9
17	25/75	PH2	6 wt%	mGGBFS	WG2.2	43.5	944.7
18	75/25	PH4	10 wt%	cGGBFS	WG2.2	18.5	975.9
19	25/75	PH2	6 wt%	cGGBFS	WG1.0	61.5	947.6
20	25/75	PH4	6 wt%	mGGBFS	WG2.2	100.5	930.6
21	25/75	PH2	10 wt%	mGGBFS	WG2.2	40.0	942.5
22	75/25	PH2	10 wt%	mGGBFS	WG1.0	33.5	969.5
23	50/50	PH3	12 wt%	cGGBFS	WG1.0	114.0	950.4
24	75/25	PH2	6 wt%	mGGBFS	WG2.2	9.0	974.5
25	0/100	PH3	8 wt%	fGGBFS	WG2.2	101.5	931.0
26	75/25	PH4	6 wt%	mGGBFS	WG1.0	54.5	939.0
27	50/50	PH5	8 wt%	fGGBFS	WG1.0	131.5	943.3
28	50/50	PH1	8 wt%	mGGBFS	WG1.0	33.0	944.7
29	50/50	PH3	12 wt%	fGGBFS	WG2.2	28.0	949.0
30	25/75	PH4	10 wt%	cGGBFS	WG2.2	186.0	933.4
31	75/25	PH2	10 wt%	cGGBFS	WG2.2	14.0	987.2
32	75/25	PH4	6 wt%	cGGBFS	WG2.2	19.5	973.0
33	50/50	PH3	8 wt%	fGGBFS	WG1.0	45.5	934.8
34	50/50	PH3	12 wt%	mGGBFS	WG2.2	42.0	949.0
35	25/75	PH2	10 wt%	cGGBFS	WG1.0	71.0	947.6
36	75/25	PH2	6 wt%	cGGBFS	WG1.0	47.0	960.3
37	25/75	PH4	6 wt%	cGGBFS	WG1.0	229.0	929.1
38	50/50	PH5	8 wt%	mGGBFS	WG2.2	70.0	937.6
39	25/75	PH4	10 wt%	mGGBFS	WG1.0	169.0	927.7
40	50/50	PH3	4 wt%	fGGBFS	WG2.2	19.0	944.7
41	100/0	PH3	8 wt%	fGGBFS	WG2.2	-	1009.9
42	50/50	PH3	8 wt%	fGGBFS	WG2.2	27.0	947.5
